# Glycoprotein A33 deficiency: a new mouse model of impaired intestinal epithelial barrier function and inflammatory disease

**DOI:** 10.1242/dmm.019935

**Published:** 2015-08-01

**Authors:** Benjamin B. Williams, Niall C. Tebbutt, Michael Buchert, Tracy L. Putoczki, Karen Doggett, Shisan Bao, Cameron N. Johnstone, Frederick Masson, Frederic Hollande, Antony W. Burgess, Andrew M. Scott, Matthias Ernst, Joan K. Heath

**Affiliations:** 1 The Walter and Eliza Hall Institute of Medical Research, Parkville, Victoria 3052, Australia; 2 Department of Medical Biology, University of Melbourne, Parkville, Victoria 3052, Australia; 3 Ludwig Institute for Cancer Research, Melbourne-Parkville Branch, Parkville, Victoria 3050, Australia; 4 Ludwig Institute for Cancer Research, Melbourne-Austin Branch, Heidelberg, Victoria 3084, Australia; 5 Discipline of Pathology, School of Medical Science and Bosch Institute, University of Sydney, Camperdown, NSW 2006, Australia; 6 Trescowthick Research Laboratories, Peter MacCallum Cancer Centre, East Melbourne, Victoria 3002, Australia; 7 Department of Pathology, University of Melbourne, Parkville, Victoria, Australia

**Keywords:** GPA33, Intestinal permeability, Colitis, Colorectal cancer, Oral tolerance

## Abstract

The cells of the intestinal epithelium provide a selectively permeable barrier between the external environment and internal tissues. The integrity of this barrier is maintained by tight junctions, specialised cell-cell contacts that permit the absorption of water and nutrients while excluding microbes, toxins and dietary antigens. Impairment of intestinal barrier function contributes to multiple gastrointestinal disorders, including food hypersensitivity, inflammatory bowel disease (IBD) and colitis-associated cancer (CAC). Glycoprotein A33 (GPA33) is an intestinal epithelium-specific cell surface marker and member of the CTX group of transmembrane proteins. Roles in cell-cell adhesion have been demonstrated for multiple CTX family members, suggesting a similar function for GPA33 within the gastrointestinal tract. To test a potential requirement for GPA33 in intestinal barrier function, we generated *Gpa33^−/−^* mice and subjected them to experimental regimens designed to produce food hypersensitivity, colitis and CAC. *Gpa33^−/−^* mice exhibited impaired intestinal barrier function. This was shown by elevated steady-state immunosurveillance in the colonic mucosa and leakiness to oral TRITC-labelled dextran after short-term exposure to dextran sodium sulphate (DSS) to injure the intestinal epithelium. *Gpa33^−/−^* mice also exhibited rapid onset and reduced resolution of DSS-induced colitis, and a striking increase in the number of colitis-associated tumours produced by treatment with the colon-specific mutagen azoxymethane (AOM) followed by two cycles of DSS. In contrast, *Gpa33^−/−^* mice treated with AOM alone showed no increase in sporadic tumour formation, indicating that their increased tumour susceptibility is dependent on inflammatory stimuli. Finally, *Gpa33^−/−^* mice displayed hypersensitivity to food allergens, a common co-morbidity in humans with IBD. We propose that *Gpa33^−/−^* mice provide a valuable model to study the mechanisms linking intestinal permeability and multiple inflammatory pathologies. Moreover, this model could facilitate preclinical studies aimed at identifying drugs that restore barrier function.

## Abstract (precis)

**Highlighted Article:** We show that GPA33, an intestine-specific cell surface protein, plays a role in the maintenance of intestinal barrier function and the prevention of intestinal pathologies such as food hypersensitivity, inflammatory bowel disease and colitis-associated cancer.

## INTRODUCTION

Impaired intestinal epithelial barrier function is an emerging factor in the aetiology and pathology of a range of gastrointestinal disorders (Turner, 2009), including food hypersensitivity (Ventura et al., 2006), inflammatory bowel disease (IBD) (Heller et al., 2005; Zeissig et al., 2007) and colitis-associated cancer (CAC) (Kim and Kim, 2014). It is becoming clear that gastrointestinal homeostasis depends on the capacity of the epithelial lining to exclude luminal bacterial and food antigens from the lamina propria (LP) and thereby prevent local and systemic inflammation. Accordingly, restoration of barrier function is an important therapeutic ambition for the treatment and prevention of inflammation-mediated pathologies (Fries et al., 2013).

The intestinal barrier is maintained by a number of interacting components: production of mucus and anti-microbial peptides, epithelial integrity, cell-cell adhesion and innate immune responses – defects in any of which can increase intestinal permeability (Pastorelli et al., 2013). Cell-cell adhesion within the intestinal epithelium is regulated by tight junctions (TJs), the most apical of the junction complexes, which block the passage of luminal contents via a paracellular route between cells. TJs are composed of a number of proteins, including occludins, claudins and members of the cortical thymocyte marker for *Xenopus* (CTX) group of type I transmembrane proteins of the immunoglobulin (Ig) superfamily (Chrétien et al., 1998). The CTX group includes members of the junction adhesion molecule (JAM) (Laukoetter et al., 2007; Vetrano and Danese, 2009; Vetrano et al., 2008), the Coxsackie and adenovirus receptor (CAR) (Morton et al., 2013; Raschperger et al., 2006) and the endothelial cell-selective adhesion molecule (ESAM) (Nasdala et al., 2002) families. Notably, the loss of JAM-A has been implicated in changes to intestinal permeability with subsequent inflammation, cytokine production and colitis in mice (Laukoetter et al., 2007) and IBD in humans (Vetrano and Danese, 2009; Vetrano et al., 2008).

A relatively understudied founding member of the CTX family (Chrétien et al., 1998) is glycoprotein A33 (GPA33) (Heath et al., 1997). In human and mouse, the expression pattern of GPA33 is exquisitely restricted to epithelial cells lining the entire length of the intestine. Within this compartment, GPA33 is present on the basolateral membranes of the epithelial cells of both absorptive and secretory lineages, with notable enrichment of GPA33 protein evident at cell-cell junctions in whole-mount preparations of colonic epithelium (Johnstone et al., 2000). Its robust expression from the base of the crypts to the tips of the villi and along the entire rostrocaudal axis of the intestinal tract established GPA33 as a definitive marker of the intestinal epithelium (Heath et al., 1997; Johnstone et al., 2000). Clinically, GPA33 expression has been observed in over 95% of colorectal cancers (CRCs) and associated metastases (Garinchesa et al., 1996), an observation that has underpinned an ongoing and concerted effort to use radionuclide-bound murine (Welt et al., 1994, 1990) and humanised (huA33 mAb) monoclonal antibodies to target GPA33-expressing tumours (Ciprotti et al., 2014; Infante et al., 2013; Scott et al., 2005; Welt et al., 2003, 1996). A recent clinical study established the efficacy of the huA33 mAb in penetrating CRC metastases (Ciprotti et al., 2014), promoting continued interest in GPA33 as a therapeutic target in antibody-based treatment of late-stage CRC.

**TRANSLATIONAL IMPACT**

**Clinical issue**

Intestinal permeability is recognised as an aetiological and pathological factor in a number of intestinal disorders. These include food hypersensitivity, inflammatory bowel disease (IBD) and colitis-associated cancer (CAC), which commonly occur as co-morbidities. The two main forms of IBD, Crohn's disease (CD) and ulcerative colitis (UC), are chronic conditions that increase the lifetime risk of colorectal cancer development 4- to 10-fold over the unaffected population and lower the age of onset by about 20 years. At present, anti-inflammatory agents make up the main treatments for IBD. However, restoration of barrier function would be an attractive therapeutic option for the treatment of IBD and prevention of CAC development. To achieve this goal, model systems with impaired intestinal barrier function and susceptibility to food hypersensitivity, in which IBD and CAC can be experimentally induced, are required. Such models offer the potential to study the mechanistic link between intestinal permeability and inflammatory disorders and to test novel therapeutic agents for the restoration of barrier function in multiple disease states.

**Results**

In this study, the authors characterised a knockout mouse with no expression of the intestinal epithelium-specific glycoprotein A33 (GPA33). *Gpa33*^–/–^ mice exhibit elevated intestinal immunosurveillance in the normal state, indicating a mild increase in intestinal permeability. This is markedly increased by environmental challenge with dextran sodium sulphate (DSS), an intestinal luminal irritant. Using this mouse model, the authors investigated the effect of impaired intestinal barrier function on a range of inducible intestinal pathologies. *Gpa33*^–/–^ mice exhibited hypersensitivity to food antigens and a markedly increased severity of colitis upon exposure to DSS. Coupled with administration of azoxymethane, a colon-specific mutagen, *Gpa33*^–/–^ DSS-treated mice developed severe CAC. These observations indicate a fundamental role for impaired barrier function in the susceptibility of *Gpa33*^–/–^ mice to a range of related inflammatory intestinal pathologies. Moreover, the analysis of publicly available gene expression data revealed that *GPA33* RNA expression is reduced in the inflamed bowel of individuals with either CD or UC, suggesting that GPA33 deficiency might contribute to intestinal permeability in human disease.

**Implications and future directions**

This study demonstrates a non-redundant role for GPA33 in the maintenance of intestinal barrier function. Future work will determine whether this function is achieved through interactions of GPA33 with components of junctional complexes that promote cell-cell adhesion in the intestine. The evidence that loss of GPA33 contributes to increased severity of multiple intestinal disorders associated with intestinal permeability advocates *Gpa33*^–/–^ mice as an advantageous model in which to test novel therapeutics designed to restore barrier function.

Despite the highly tissue-specific expression pattern and associated clinical interest, relatively little is known about the function of GPA33. Insights into its transcriptional regulation (Johnstone et al., 2002; Mao et al., 2003), and implied roles in cell-cell adhesion (Ackerman et al., 2008) and immune signalling (Mallegol et al., 2005, 2007; Van Niel et al., 2003), constitute the most prominent findings to date. In a prior report, conducted with *Gpa33^−/−^* mice on a mixed background (C57BL/6×129Sv/J), we noticed a mild elevation in spontaneous colonic ulceration and a more severe response to the colonic injury induced by trinitrobenzenesulfonic acid (TNBS), compared to wild-type (WT) mice (Pereira-Fantini et al., 2010).

In this study, we analysed the role of GPA33 in intestinal barrier function using *Gpa33^−/−^* mice on a pure C57BL/6 background. Our findings indicate a role for GPA33 in the maintenance of intestinal barrier function and the prevention of associated pathologies such as IBD.

## RESULTS

### *Gpa33^−/−^* mice do not exhibit spontaneous gastrointestinal pathology

**Fig. 1.**
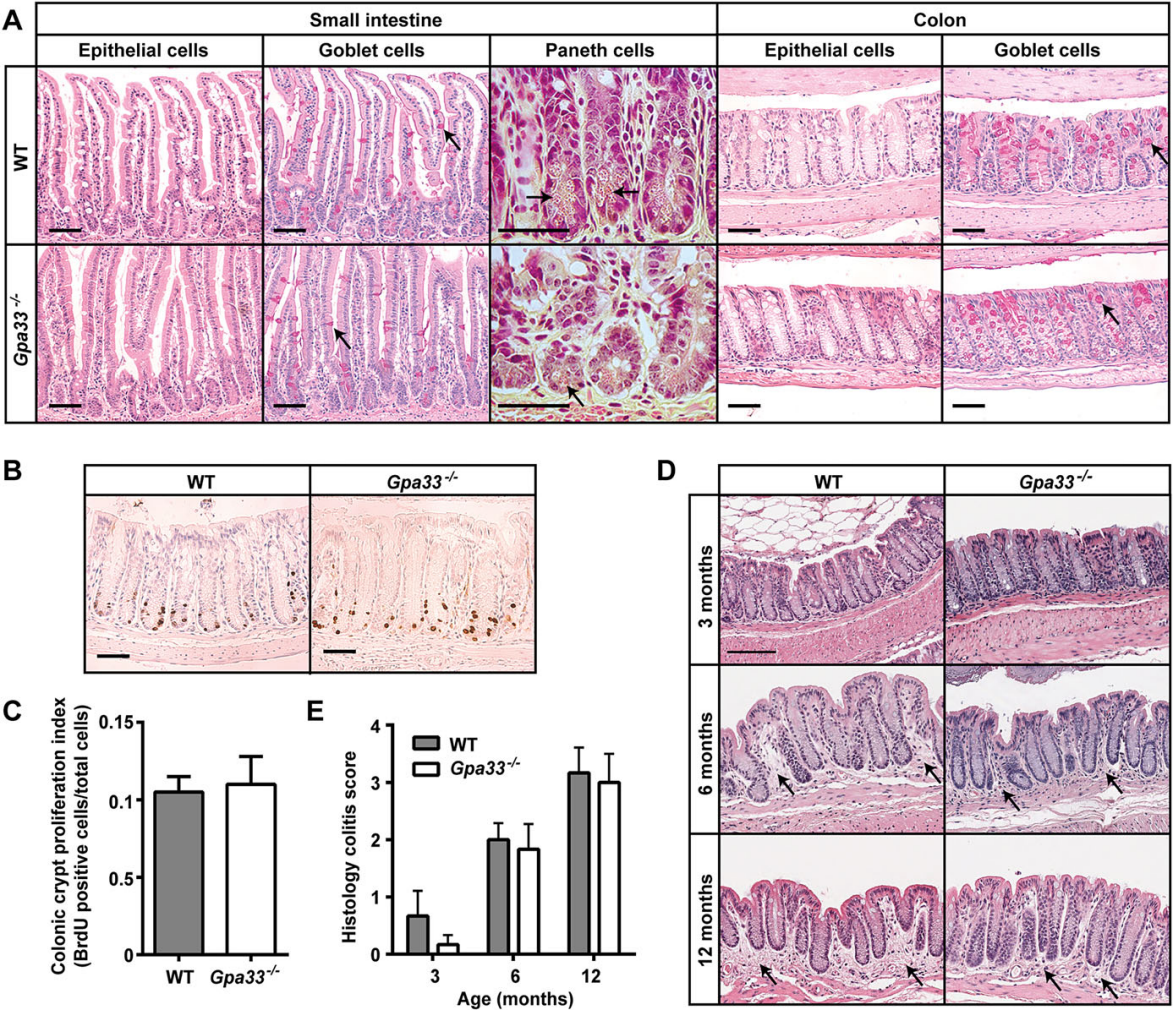
***Gpa33^−/−^* mice do not exhibit steady-state gastrointestinal pathology.** (A) Representative histological images of small intestine and colon from 10-week-old *Gpa33^−/−^* and WT mice on a pure C57Bl/6 background. H&E stains all epithelial cells, Periodic acid-Schiff stains the mucins in goblet cells pink (arrows), and phloxine and tartrazine highlights the granules in Paneth cells (arrows). (B) No difference in proliferation is observed in colonic crypts of *Gpa33^−/−^* and WT mice. Representative BrdU immunohistochemistry in the colon of 10-week-old *Gpa33^−/−^* and WT mice collected 2 h after intraperitoneal BrdU injection (100 mg/kg). (C) Quantitation of BrdU-positive cells expressed as a fraction of total cells per crypt. A minimum of ten crypts were evaluated per mouse. (D) Both *Gpa33^−/−^* and WT mice exhibit mild progressive colitis with age. Representative images of H&E-stained distal colon sections from mice aged 3, 6 and 12 months. Arrows indicate sub-mucosal thickening and immune cell infiltration, characteristic of colitis. (E) Quantitation of colitis using the scheme shown in supplementary material Table S1. Mean±s.e.m., *n*=3. Scale bars: 50 µm.

A null allele of *Gpa33* was generated by replacing most of the *Gpa33* coding sequence with a promoter-less *β-geo* cassette as described in supplementary material Fig. S1A. Expression of GPA33 was clearly detectable in the intestinal epithelium of WT mice and undetectable in *Gpa33^−/−^* mice, as shown by RT-qPCR (supplementary material Fig. S1B) and immunohistochemistry (supplementary material Fig. S1C), confirming the allele as a bona fide null. *Gpa33^−/−^* mice were born at the expected frequency, are fertile and have a normal lifespan. Intestinal epithelial cell lineage differentiation (Fig. 1A) and proliferation (Fig. 1B,C) were found to be normal in *Gpa33^−/−^* mice and, apart from an extremely mild, progressive colitis with age, which is indistinguishable from that seen in WT, *Gpa33^−/−^* mice did not exhibit steady-state colitis (Fig. 1D,E).

### *Gpa33^−/−^* mice have impaired intestinal barrier function

**Fig. 2.**
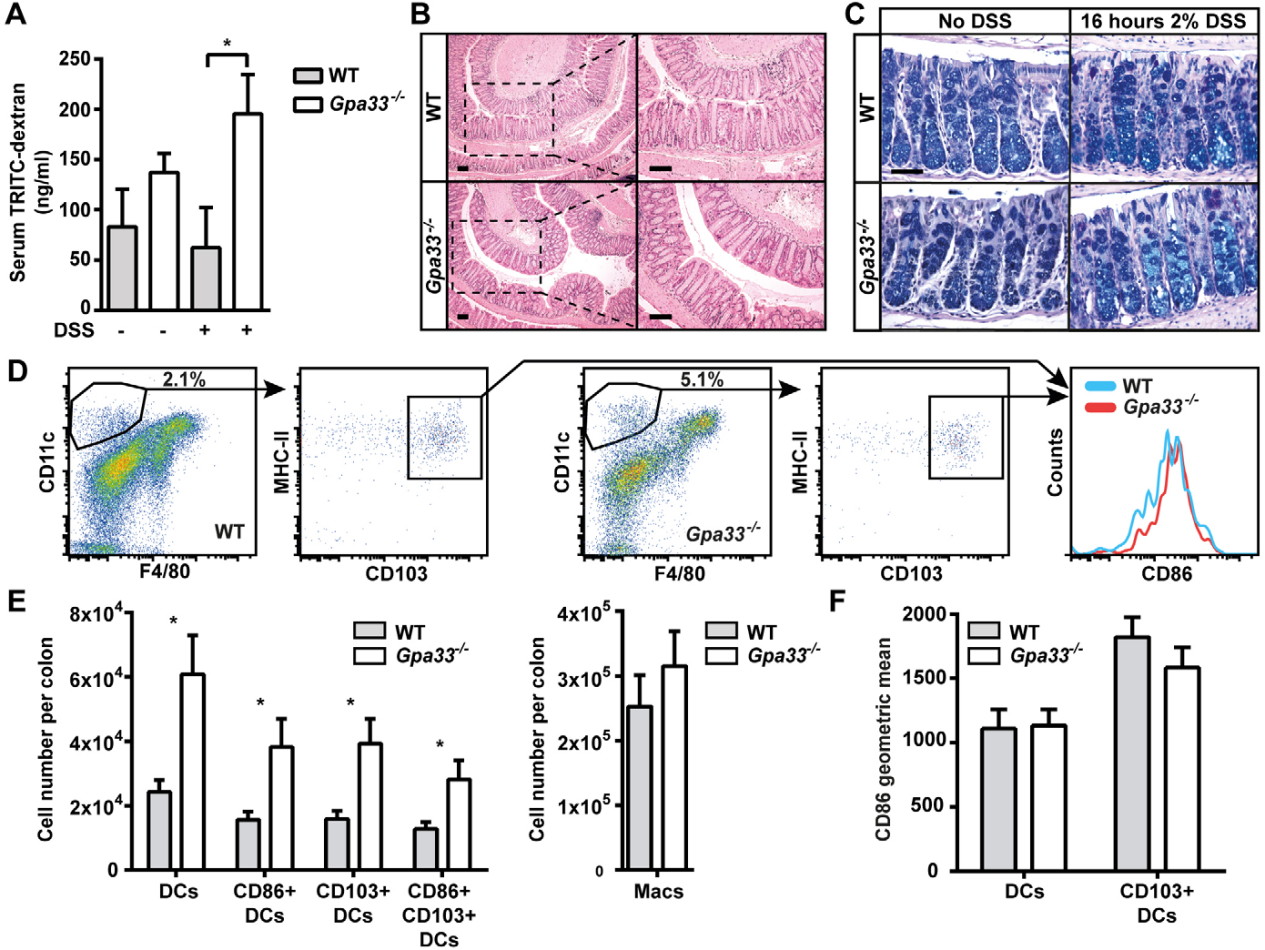
***Gpa33^−/−^* mice are sensitised to impaired intestinal barrier function.** (A) Mice were provided with 2% DSS in the drinking water *ad libitum* for 16 h following which TRITC-dextran was administered by gavage. Four hours later, the TRITC signal in the serum was quantitated as a measure of intestinal permeability. DSS-treated *Gpa33^−/−^* but not WT mice exhibit an increase in serum TRITC-dextran, which is indicative of impaired intestinal barrier function. (B) Representative images of H&E-stained distal and mid colon sections from mice treated with 2% DSS in the drinking water for 16 h, illustrating morphological integrity of the epithelial layer after DSS treatment. The right-hand column provides higher magnifications of the boxed areas. (C) Representative histological images of mid colon from 10-week-old *Gpa33^−/−^* and WT mice stained with alcian blue and Periodic acid-Schiff for neutral (magenta) and acidic (blue) mucins, respectively, in the presence and absence of DSS treatment. Scale bars: (B) 100 µm and (C) 50 µm. (D) Single-cell suspensions of colon LP were prepared and the numbers of DCs and macrophages were determined by FACS. The gating strategy for activated CD103^+^ DCs derived from viable, single, haematopoietic cells (Epcam^−^ CD45^+^) is shown. (E) Absolute cell numbers for populations within the whole colon LP were calculated using relative cell populations obtained by FACS and viable single-cell counts. *Gpa33^−/−^* mice exhibit an increase in activated DCs within the colonic LP relative to WT controls, indicating elevated immunosurveillance. Populations represent: DCs (Epcam^−^ CD45^+^ CD11c^+^ F4/80^−^ MHC-II^+^), CD86^+^ activated DCs, gut resident CD103^+^ DCs and macrophages (Epcam^−^ CD45^+^ CD11c^+^ F4/80^+^). (F) Geometric mean for CD86 in DCs and CD130^+^ DC populations. Mean±s.e.m., (A) *n*=7-8, (E,F) *n*=6, *P<0.05.

To study the requirement for GPA33 in the maintenance of intestinal barrier function, we delivered TRITC-labelled dextran (4 kDa) to the gastrointestinal tract by gavage and measured its levels in the circulation 4 h later. WT and *Gpa33^−/−^* mice did not exhibit significant, detectable intestinal permeability to dextran in the steady-state. To test barrier function under challenge conditions, the experiment was repeated with a cohort of mice that received 2% dextran sodium sulphate (DSS) in the drinking water for 16 h prior to TRITC-dextran gavage (Brandl et al., 2009). This short-term DSS treatment did not cause morphological epithelial damage in the colon of either genotype (Fig. 2B,C). WT mice were unaffected by 2% DSS treatment, in accord with a published report in which effects on intestinal permeability were first observed in WT mice only after 3 days of 5% DSS treatment (Kitajima et al., 1999). In contrast, after DSS treatment, *Gpa33^−/−^* mice demonstrated a more than threefold increase in the level of serum TRITC-dextran (Fig. 2A) over WT mice exposed to DSS, indicating a relative loss of barrier function in *Gpa33^−/−^* mice compared to WT.

In order to further interrogate steady-state barrier function in *Gpa33^−/−^* mice, dendritic cells (DCs) within the colonic LP were analysed by flow cytometry. A range of DC populations were elevated in the colonic LP (Fig. 2E) but not the spleen (supplementary material Fig. S2B) of *Gpa33^−/−^* mice, most notably activated DCs and activated CD103^+^ gut-resident DCs (Fig. 2D,E). Although more total DCs were present in *Gpa33^−/−^* mice, the relative activation state of DC populations in the colonic LP were similar in *Gpa33^−/−^* and WT mice (Fig. 2D,F). This elevated immunosurveillance phenotype is not dependent on *Gpa33* expression in haematopoietic cells: *Gpa33* expression was undetectable in 39 blood cell populations encompassing both myeloid and lymphoid lineages (supplementary material Fig. S3). Collectively, these data suggest that *Gpa33^−/−^* mice exhibit an intestinal epithelial cell-intrinsic mild and sub-pathological intestinal permeability (IP) defect that sensitises them to intestinal challenge.

### *Gpa33^−/−^* mice are susceptible to colitis induction

**Fig. 3.**
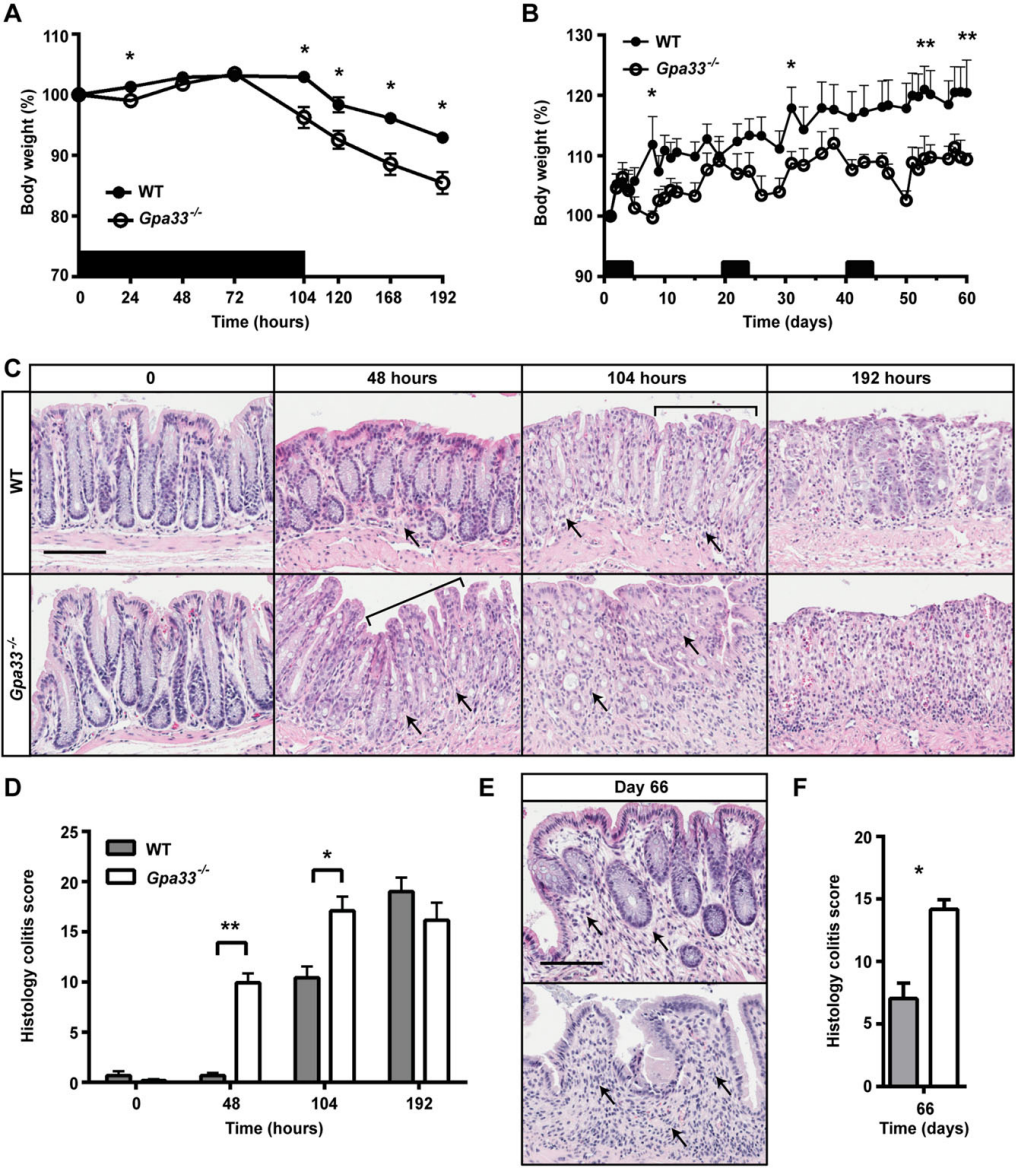
***Gpa33^−/−^* mice are more susceptible to DSS-induced colitis.** (A,B) Black bars indicate cycles of 2% DSS treatment provided *ad libitum* in drinking water. (A) Percentage body weight changes during acute colitis reveal that *Gpa33^−/−^* mice lose significantly more weight than WT mice in response to treatment with 2% DSS (asterisks) for 104 h. (B) Percentage body weight changes during chronic colitis reveal that *Gpa33^−/−^* mice lose significantly more weight than WT mice after each cycle of DSS treatment, indicated by asterisks on days 8, 31, 50 and 61. *Gpa33^−/−^* mice also exhibited significantly reduced percentage body weight on days 10, 12, 47, 51, 52 and 54 (*P*<0.05). (C) Representative images of H&E-stained distal colon sections from mice that were exposed to acute DSS treatment for 0, 48 and 104 h and after an additional 88 h of normal water (192 h time-point). Crypt degradation is evident in *Gpa33^−/−^* mice at 48 h but not in WT mice until 104 h (brackets). Arrows indicate immune cell infiltration and thickening of the LP, which is first conspicuous at 48 h in *Gpa33^−/−^* mice but not until 104 h in WT controls. (D) Histology colitis scores at 0, 48, 104 and 192 h show accelerated colitis onset in *Gpa33^−/−^* mice. Also, the histology colitis score is significantly higher at 192 h than at 104 h in WT mice (*P*<0.01). (E) Representative images of H&E-stained distal colon sections from mice (day 66) that underwent chronic DSS treatment. Arrows indicate immune cell infiltration and thickening of the LP, which is most pronounced in the *Gpa33^−/−^* mice. (F) Histology colitis score of mice at day 66 following chronic colitis regimen. Mean±s.e.m. for A,B,D,F; *n*=5 (A), *n*=5-8 (B), *n*=3 (D), *n*=4 (F); *P<0.05, **P<0.01. Scale bars: 50 µm.

To investigate the effect of GPA33 deficiency on susceptibility to colitis induction, we treated *Gpa33^−/−^* mice with 2% DSS. Two different DSS treatment regimens were employed to induce acute or chronic colitis. In the acute colitis model, mice were exposed to 2% DSS in the drinking water *ad libitum* for 104 h and then switched to normal drinking water for a further 88 h (192 h in total). *Gpa33^−/−^* mice exhibited a more rapid onset of colitis compared to WT mice, shown by increased weight loss (Fig. 3A), and histological damage (Fig. 3C,D). Maximal colitis in *Gpa33^−/−^* mice was observed at 104 h, whereas the colitis score significantly increased between 104 h and 192 h (*P*<0.01) in WT mice (Fig. 3C,D), demonstrating accelerated onset and progression of DSS-induced colitis in *Gpa33*-deficient animals. At the 192 h time point, both genotypes exhibited similar colitis severity, suggesting that *Gpa33* deficiency mostly affects colitis onset (Fig. 3C,D). In the chronic colitis model, mice were exposed to three cycles of 2% DSS in the drinking water for 5 days followed by normal drinking water for 16 days. *Gpa33^−/−^* mice also exhibited increased susceptibility to chronic colitis as shown by more exaggerated weight loss following each cycle of DSS (Fig. 3B) and impaired crypt regeneration and resolution of colonic inflammation compared to WT (Fig. 3E,F). Together, these observations demonstrate rapid onset of acute colitis and delayed resolution of chronic colitis in *Gpa33^−/−^* mice.

### *Gpa33^−/−^* mice are susceptible to colitis-associated cancer (CAC)

**Fig. 4.**
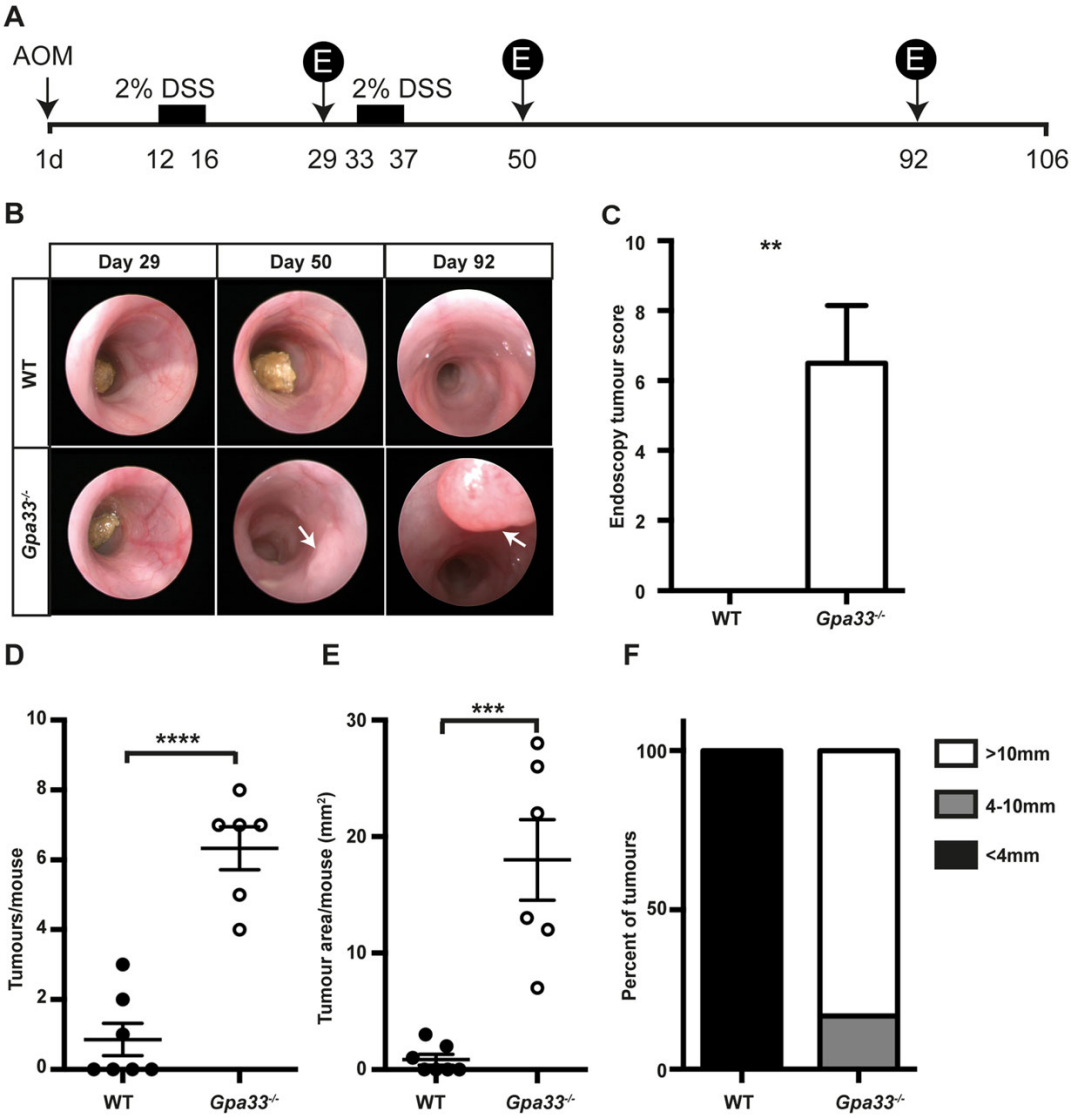
***Gpa33^−/−^* mice are more susceptible to colitis-associated cancer (CAC).** (A) Schematic representation of the regimen used to induce CAC, whereby a single AOM injection (10 mg/kg body weight) is followed by two cycles of 2% DSS provided *ad libitum* in the drinking water. Tumour development was monitored by endoscopy at the indicated times (days) post-AOM treatment (denoted by ‘E’ in the schematic). (B) Representative endoscopy images indicating the presence of tumours (arrows) in the distal portion (final 2.5 cm) of the colon in *Gpa33^−/−^* mice at day 50 and day 92 post-AOM injection. (C) Endoscopy tumour score at day 92 post-AOM administration indicates that *Gpa33^−/−^* mice exhibit increased incidence of CAC. (D,E) At collection (106 days post-AOM injection), tumour number (D) and total tumour area (E) per mouse are elevated in *Gpa33^−/−^* mice compared to WT mice. (F) The mean area of tumours in WT mice is less than 4 mm^2^, whereas in *Gpa33^−/−^*mice a large proportion (>80%) of tumours are greater than 10 mm^2^ in area. Mean±s.e.m.; *n*=6-7 (C-F); **P<0.005, ***P<0.0005, ****P<0.0001.

Chronic colitis is a susceptibility factor for CRC (Eaden et al., 2001; Kim and Chang, 2014). To test whether the rapid induction and impaired resolution of colitis in *Gpa33^−/−^* mice leads to increased development of CAC, we employed the azoxymethane (AOM)/DSS model (Neufert et al., 2007; Wirtz et al., 2007). Mice were injected with the colon-specific mutagen AOM and subjected to two cycles of 2% DSS in drinking water to induce chronic colitis (Fig. 4A). Colon tumour burden was markedly elevated in *Gpa33^−/−^* mice compared to WT mice, as shown by endoscopy of the most distal part of the colon in live animals (Fig. 4B,C) and by quantitating the number and area of the tumours at the end of the experiment (Fig. 4D-F). Both WT and *Gpa33^−/−^* mice exhibited activation of the WNT pathway, shown by elevated β-catenin protein (supplementary material Fig. S4A) and upregulated expression of the WNT target genes, *CD44* and *Lgr5*, in tumours compared to normal epithelium (supplementary material Fig. S4B), consistent with *CTNNB* (encoding β-catenin) being a known AOM target.

### *Gpa33^−/−^* mice are not predisposed to sporadic CRC

**Fig. 5.**
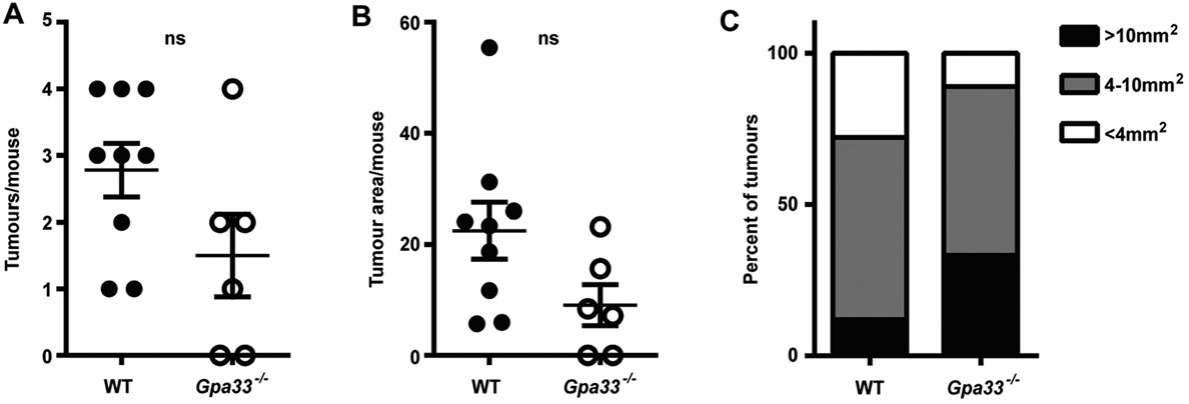
***Gpa33^−/−^* mice are not prone to sporadic CRC.** Mice were injected with AOM (10 mg/kg body weight) six times at weekly intervals to induce sporadic CRC. (A) Tumour number and (B) tumour area per mouse colon at collection was similar in *Gpa33^−/−^* and WT mice. (C) Size distribution of sporadic CRC tumours across all *Gpa33^−/−^* and WT mice at the end of the experiment. Mean±s.e.m.; *n*=6-9.

To investigate the requirement for inflammation in the susceptibility of *Gpa33^−/−^* mice to CAC, a model of sporadic CRC was employed. Mice were injected weekly with AOM alone (10 mg/kg) for 6 weeks and aged until 18 weeks to allow outgrowth of induced lesions. In this model, the absence of DSS removes the major inflammatory driver of tumorigenesis present in the AOM/DSS CAC model. *Gpa33^−/−^* mice did not exhibit an increase in sporadic CRC incidence (Fig. 5A) or tumour size (Fig. 5B,C) compared to WT controls. This finding indicates that inflammation is required for the increased susceptibility of *Gpa33^−/−^* mice to colonic tumorigenesis.

### *Gpa33^−/−^* mice exhibit increased sensitivity to food antigen

**Fig. 6.**
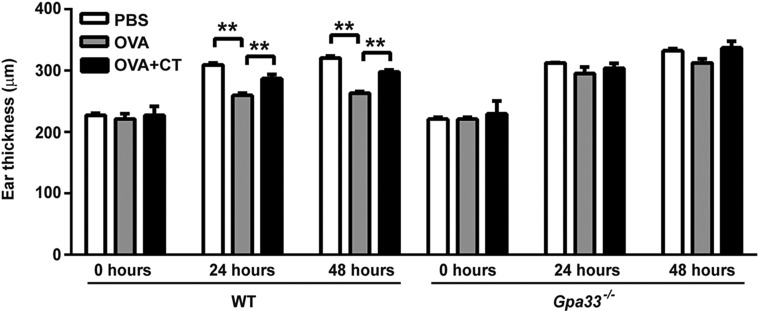
***Gpa33^−/−^* mice lack tolerance to oral antigen.** *Gpa33^−/−^* mice exhibit lack of oral tolerance to OVA administered by gavage, with resultant systemic delayed-type hypersensitivity (DTH) response manifested as increased ear thickness at the topical OVA injection site. Administration by gavage of PBS, or OVA in combination with cholera toxin (OVA+CT), make up the positive controls for lack of oral tolerance. Whereas WT mice gavaged with OVA alone exhibit oral tolerance compared to WT mice treated with PBS and OVA+CT (mean±s.d., *n*=6-7, **P<0.01), *Gpa33^−/−^* mice gavaged with OVA exhibit increased ear thickness comparable to positive controls, indicative of lack of oral tolerance and systemic DTH. Compared to baseline levels of ear thickness at the time of local OVA injection (0 h), the percentage increases in ear thickness of *Gpa33^−/−^* mice at 24 h and 48 h are 33% (*P*<0.0001) and 41% (*P*<0.0001), respectively.

A clinical consequence of increased IP is the loss of tolerance to food antigens, with increasing severity of hypersensitivity correlated with diminishing barrier function (Ventura et al., 2006). To examine delayed-type hypersensitivity (DTH) to oral ovalbumin (OVA) in *Gpa33^−/−^* and WT controls, mice were gavaged with OVA to induce tolerance, injected intraperitoneally 7 days later with OVA and Freund's complete adjuvant to boost any primed immune response, and injected with OVA in the ear to elicit local inflammation and changes in ear thickness (Wang et al., 2007). Ear thickness was measured with a calliper at 0, 24 and 48 h after intra-ear injection of OVA. In the positive control cohorts, which were gavaged with PBS alone, or OVA in combination with cholera toxin (OVA+CT) to break tolerance to OVA, topical injection of OVA caused an increase in ear thickness 24 and 48 h later. The development of DTH and broken oral tolerance in these mice occurred irrespective of genotype, as expected (Fig. 6). For WT mice that had been gavaged with OVA alone, the mean ear thickness was significantly less than that of the PBS and OVA+CT controls, indicating that oral tolerance had been established. In contrast, the mean ear thickness in *Gpa33^−/−^* mice that had been gavaged with OVA alone was not significantly different from that of *Gpa33^−/−^* mice that had been gavaged with PBS, or OVA+CT, indicating systemic hypersensitivity to OVA and broken oral tolerance (Fig. 6).

### *GPA33* expression is reduced in the inflamed bowel of individuals with IBD

**Fig. 7.**
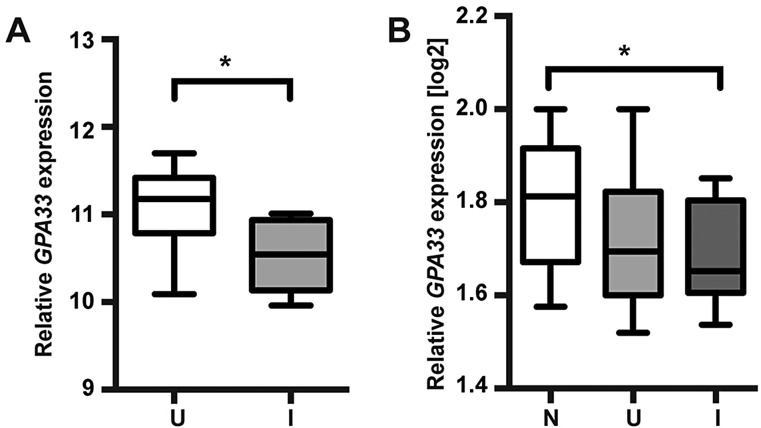
***GPA33* expression is reduced in the inflamed bowel of individuals with IBD.** Relative *GPA33* expression in normal (N), uninflamed (U) and inflamed (I) bowel of individuals with IBD represented as whisker and box plots; *P<0.05. (A) *GPA33* expression is elevated in inflamed compared to uninflamed bowel from individuals with CD (uninflamed *n*=18, inflamed *n*=8; dataset GDS3119) (Olsen et al., 2009). (B) *GPA33* expression is elevated in inflamed bowel from individuals with UC compared to normal bowel (normal *n*=22, uninflamed *n*=15, inflamed *n*=19; dataset GDS3268) (Noble et al., 2008). Relative gene expression values were normalised using (A) Human Genome U133 Plus 2.0 Array Normalisation Controls (Affymetrix) and (B) Stratagene Universal Human Reference (Stratagene).

In order to investigate the potential role of GPA33 deficiency in human IBD, *GPA33* expression was analysed in two published human IBD microarray datasets (Noble et al., 2008; Olsen et al., 2009). In individuals with either Crohn's disease (CD) or ulcerative colitis (UC), mean *GPA33* expression was reduced in inflamed bowel compared to uninflamed and normal control tissue, respectively (Fig. 7). To address the possibility that the reduction in the mean level of *GPA33* expression is related to the epithelial cell content of the inflamed samples, we also analysed the expression of *EPCAM*, an epithelial marker that is also highly expressed in intestinal epithelium. We found that *EPCAM* was not differentially expressed between uninflamed and inflamed tissue in both the CD and UC datasets (supplementary material Fig. S5A,B). We also analysed the expression of *FAP* (encoding fibroblast activation protein, expressed in activated stromal fibroblasts of epithelial cancers and healing wounds) and found that its expression was unchanged between uninflamed and inflamed tissues from individuals with CD and elevated in the inflamed tissues of individuals with UC (supplementary material Fig. S5C,D). Analysis of the expression of an additional epithelial marker (*CDH1*, encoding E-cadherin) and another stromal marker (*VIM*, encoding vimentin) reiterated the *EPCAM* and *FAP* results, respectively (not shown). Collectively, these data suggest that the observed reduction in mean *GPA33* expression in the inflamed tissue of individuals with CD or UC is not simply due to reduced epithelial cell content of the samples. These data suggest that *GPA33* deficiency correlates with intestinal inflammation in IBD and that it might contribute to IP in this context.

## DISCUSSION

*Gpa33^−/−^* mice on a pure C57BL/6 background are healthy and do not develop spontaneous colitis or any other gastrointestinal pathology up to 1 year of age. Strikingly, however, *Gpa33^−/−^* mice are more susceptible than WT to DSS-induced loss of intestinal barrier function and severe colitis and, when DSS treatment is coupled with prior administration of the mutagen AOM, they develop significantly more and larger colon tumours. Further evidence that GPA33 plays a role in barrier function is the observation that *Gpa33^−/−^* mice fail to establish tolerance to the oral antigen OVA and develop DTH.

To investigate the contribution of GPA33 to intestinal barrier integrity, we assessed immunosurveillance in the colon and paracellular penetration of 4 kDa dextran in WT and *Gpa33^−/−^* mice. Although leakiness to dextran was not significantly elevated in *Gpa33^−/−^* mice under steady-state conditions, *Gpa33^−/−^* mice exhibited a twofold increase in activated CD103^+^ DCs, the major class of antigen-presenting cells required for induction both of tolerance to benign antigen and inflammation to clear pathogens (Scott et al., 2011). This observation suggests that *Gpa33^−/−^* mice exhibit a mild increase in luminal antigen flux into the colonic LP.

To analyse the pathological consequences of increased IP and inflammation on *Gpa33^−/−^* mice, we exploited several DSS models of colitis (Wirtz et al., 2007; Wirtz and Neurath, 2007). Short-term DSS treatment (2% DSS for 16 h) reduces mucus protection of the most luminal epithelial cells, and allows penetration of bacteria closer to the epithelium (Johansson et al., 2010). Although this did not affect epithelial cell integrity or induce paracellular permeability in WT mice, which is consistent with previous findings (Kitajima et al., 1999), *Gpa33^−/−^* mice exhibited dextran permeability upon mucus layer degradation, indicating an underlying defect in cell-cell adhesion. Similar findings have been reported for mice individually deficient for CAR and Occludin, indicating that a number of junctional proteins might play non-redundant roles in the steady-state maintenance of TJs and barrier function (Pazirandeh et al., 2011; Saitou et al., 2000).

Using a harsher regime of DSS treatment (2% DSS in drinking water for 104 h), *Gpa33^−/−^* mice exhibited a rapid onset of acute colitis, indicative of luminal antigen penetration into the LP, most likely caused by TJ impairment and enhanced paracellular permeability. Similar observations have been reported for DSS-treated *Jam-A*-knockout mice, demonstrating a direct link between TJ dysfunction and severe colitis (Laukoetter et al., 2007; Vetrano et al., 2008). When exposed to repeated DSS cycles to induce chronic colitis akin to the flares of inflammation in IBD, *Gpa33^−/−^* mice were less able to resolve multiple bursts of epithelial damage and inflammation than WT controls. Considered in conjunction with the reduced *GPA33* expression observed in the inflamed bowel of humans with either CD or UC, this suggests a potential role for GPA33 impairment in the progression of IBD.

Individuals with IBD have an increased risk of developing CRC (Eaden et al., 2001; Itzkowitz and Yio, 2004; Kim and Kim, 2014). This fundamental role for inflammation in CRC is modelled using AOM/DSS treatment of mice to induce CAC (Neufert et al., 2007). Strikingly, *Gpa33^−/−^* mice develop increased numbers of and larger tumours than WT control mice, suggesting that loss of GPA33 leads to both an increase in the incidence of tumour formation and the rate of tumour growth. These observations are consistent with increased inflammation and cytokine production stimulating outgrowth of AOM-mutated tumour-initiating cells in *Gpa33^−/−^* mice (Wirtz et al., 2007). Meanwhile, induction of sporadic CRC in *Gpa33^−/−^* mice by injection with AOM alone did not result in elevated tumorigenesis, confirming the essential requirement for inflammation and the underlying loss of permeability as the fundamental defect.

The pathological consequences of IP are not restricted to IBD and localised inflammation in the intestinal tract, but can also lead to loss of oral tolerance and systemic inflammatory effects. A leaky epithelium can allow luminal contents including food antigens to bypass normal antigen processing and initiate an inflammatory response in the LP with consequent systemic sensitisation to that food antigen (Wirtz and Neurath, 2007). Indeed, food hypersensitivity can precede IBD onset and is a recognised co-morbidity (Kraus et al., 2004). Using an established OVA model of oral DTH (Wang et al., 2007) we observed an increased response in *Gpa33^−/−^* mice, providing further evidence for IP in GPA33-deficient contexts.

In addition to the results presented here, and the demonstration that other CTX family members, such as JAM-A, play a role in cell-cell adhesion, *in vitro* experiments have also suggested an epithelial cell-intrinsic requirement for GPA33 in barrier formation and junctional complex integrity (Ackerman et al., 2008). Further work will establish the precise mechanism by which GPA33 acts to maintain barrier function and protect against disease, although the cumulative evidence to date points to a role in cell-cell adhesion.

The highly restricted expression of GPA33 to the intestinal epithelium and CRC is a hallmark that has sustained the interest in GPA33 as a target for antibody-based cancer therapy. Interestingly, however, Sakamoto et al. (2000) also detected weak expression of GPA33 in murine salivary glands. This observation, coupled with two studies regarding Sjögren's syndrome (SS; Ewert et al., 2010; Nguyen et al., 2008), suggests a possible extra-intestinal role for GPA33 in barrier function. SS is an autoimmune disorder in which the salivary and lacrimal glands are destroyed by infiltrating lymphocytes, leading to symptomatic dry mouth and eyes in affected individuals. Although the aetiology of this disorder is thought to be polygenic, a recent study implicated TJ disruption and resultant pro-inflammatory cytokine exposure within the salivary glands as a key pathogenic factor (Ewert et al., 2010). Interestingly, investigation of SS using inbred mouse models identified *Gpa33* as one of a handful of genes deleted in two SS susceptibility loci (Nguyen et al., 2008). This tantalising observation suggests that GPA33 might play a role in barrier function outside the intestine.

A number of genetically modified mouse models with deranged junctional complexes and barrier dysfunction have been reported, for which a subset develop spontaneous or inducible inflammatory pathology and provide useful preclinical models of colitis and CAC (Hermiston and Gordon, 1995; Laukoetter et al., 2007; Rudolph et al., 1995; Saitou et al., 2000; Smalley-Freed et al., 2010). The ease with which *Gpa33^−/−^* mice can be manipulated to elicit a suite of intestinal pathologies, including loss of oral tolerance, severe colitis and CAC, suggests that this model might provide an attractive option for preclinical studies aimed at identifying drugs capable of restoring intestinal barrier function.

## MATERIALS AND METHODS

### Animals

All animal experiments were conducted with the approval of the animal ethics committees of The Ludwig Institute for Cancer Research, The Walter and Eliza Hall Institute of Medical Research and the University of Sydney (Australia). All mice were maintained on a pure C57BL/6 genetic background except those used to assess oral tolerance. Cohorts for the oral tolerance experiments were maintained on a mixed 129/SvJ, C57BL/6 background with littermate controls used at all times. Mice were bred and housed in the same room in a specific pathogen-free facility at the Ludwig Institute of Cancer Research to minimise variation in gut microbiota. All mice were 8 to 12 weeks old at the commencement of treatment, or when analysed, unless otherwise indicated.

### Generation of *Gpa33^−/−^* mice

We generated embryonic stem (ES) cells (129Sv/J background) containing a null *Gpa33* allele by replacing 20 kb of endogenous *Gpa33* sequence from the 5′ end of exon 2 to the 5′ end of exon 7 with a promoter-less targeting vector. The targeting vector contained a translational stop codon (TGA) at codon 35 of exon 2 to terminate translation of an encoded truncated (13 amino acid) GPA33 protein. This was followed by an internal ribosome entry site (IRES) to permit translation of a *β-geo* cassette encoding a β-galactosidase–neomycin fusion protein (supplementary material Fig. S1A) to allow selection of targeted neomycin-resistant ES cells and visualisation of *Gpa33* promoter activity *in vivo* via blue staining following X-gal metabolism. A congenic *Gpa33^−/−^* C57BL/6 strain was generated by backcrossing the *Gpa33^−/−^* 129Sv/J×C57BL/6 founder mice to C57BL/6 mice for a minimum of ten generations.

### RNA analysis

RNA was extracted from purified epithelial cell fractions harvested from freshly dissected small and large intestines (Whitehead et al., 1987) and dissected tumour tissue using TRIzol (Life Technologies, Carlsbad, CA, USA). RNA integrity was analysed on a 2100 Bioanalyzer (Agilent, Santa Clara, CA, USA) and cDNA generated using the High Fidelity cDNA synthesis kit (Applied Biosystems, Waltham, MA, USA). Quantitative RT-PCR was performed using SYBR green (Quantace, London, UK) on an ABI 7300 real-time PCR machine using primers designed to amplify *Gpa33*, *Lgr5*, *CD44* and *Hprt1* (sequences available on request). Expression data was normalised to *Hprt1* expression.

### *In vivo* assessment of intestinal permeability

Mice were exposed to either normal drinking water or DSS (2% w/v, MW 35-50 kDa; MP Biomedicals, Santa Ana, CA, USA) in drinking water *ad libitum* for 16 h prior to oral gavage with 200 µl of TRITC-dextran (40 mg/ml TRITC-dextran 4 kDa in PBS; Sigma-Aldrich, St Louis, MO, USA). Circulating TRITC signal was quantitated by fluorometry on serum collected 4 h post-gavage, as described previously (Brandl et al., 2009).

### Fluorescence-activated cell sorting (FACS) analysis

Analyses of haematopoietic populations in the colonic LP and spleen were performed by FACS on single-cell suspensions. Colon LP was prepared by incubating washed, finely chopped colon in 20 ml dissociation solution comprising FCS (2.5%; Life Technologies, Carlsbad, CA, USA), Hanks’ Balanced Salt Solution (HBSS) without Ca++ and Mg++ (Life Technologies, Carlsbad, CA, USA), EDTA (5 mM; BDH Chemicals, Radnor, PA, USA) and DTT (1 mM; Promega, Madison, WI, USA) for 30 min at 37°C with constant shaking and vortexing at 15 and 30 min to release epithelial cells. The remaining tissues were digested in 10 ml of 2.5% FCS, HBBS (Ca++- and Mg++-free), Collagenase III (1 mg/ml; Worthington, Lakewood, NJ, USA), DNaseI (200 μg/ml; Roche, Basel, Switzerland), Dispase (0.4 U/ml; Sigma-Aldrich, St Louis, MO, USA) for 45 min with vortexing every 15 min. Spleen was finely chopped and digested in 10 ml RPMI (Life Technologies, Carlsbad, CA, USA) containing 2.5% FCS and 1 mg/ml Collagenase III for 20 min at room temperature (RT) with constant agitation. Collected single cells were washed, counted and stained using mixtures of antibodies to specifically identify myeloid cell populations: Epcam-APC (47-5791; eBioscience, San Diego, CA, USA), CD45-BV421 (563890; BD Pharmingen), CD11c-BV656 (553800; BD Pharmingen), F4/80-FITC (11-4801; eBioscience, San Diego, CA, USA), MHC-II-APC-Cy7 (Walter and Eliza Hall Institute of Medical Research, Biotechnology Centre, Melbourne, Victoria, Australia), CD103-PE (557495; BD Pharmingen), CD86-PE-Cy7 (A15412; Life Technologies, Carlsbad, CA, USA) and lymphoid cell populations: Epcam-FITC (11-5791; eBioscience), CD45-BV421 (563890; BD Pharmingen), CD11b-PE (553311; BD Pharmingen), TCRβ-PerCP (557019; BD Pharmingen), B220-biotin (553086; BD Pharmingen), Streptavadin-BV650 (405231; BioLegend, San Diego, CA, USA), CD4-APC (GK1.5; Walter and Eliza Hall Institute of Medical Research, Biotechnology Centre) and CD8-PE-Cy7 (552877; BD Pharmingen). Acquisition was performed on a Fortessa X20 (BD Biosciences, San Jose, CA, USA) with viability assessed using propidium iodide (Sigma-Aldrich, St Louis, MO, USA) and data analysed using FlowJo software (FlowJo, Ashland, OR, USA). Absolute cell numbers for haematopoietic populations in colonic LP or spleen were calculated using whole organ, single, viable cell counts and the derived relative populations obtained by FACS.

### Induction of acute and chronic colitis

One cycle of colitis was induced by *ad libitum* exposure to DSS (2%; Sigma-Aldrich, St Louis, MO, USA) in drinking water for 104 h, followed by normal drinking water. In the acute colitis experiment, mice were euthanised after 0, 48 and 104 h of DSS treatment and after a further 88 h of normal water (192 h time-point). To induce chronic colitis, mice were treated with three cycles of 2% DSS interspersed with normal water for 16 days. Mice in both models were weighed daily to observe weight loss consequent to colitis. For histological analyses, the colons were opened lengthwise from anus to caecum, rolled and fixed in 10% neutral buffered formalin (Pathtech, Melbourne, Victoria, Australia).

### Colitis-associated and sporadic colon cancer

CAC was induced through a single intraperitoneal injection of AOM (10 mg/kg; Sigma-Aldrich, St Louis, MO, USA) in PBS, followed 12 days later by two cycles of DSS treatment. Both cycles comprised *ad libitum* exposure to 2% DSS for 5 days in drinking water, interspersed with 16 days of normal drinking water. To mimic sporadic CRC, mice received weekly intraperitoneal injections of AOM (10 mg/kg) for 6 weeks. Tumour burden in the colon was monitored using a Coloview high-resolution mouse video endoscopic system (Karl Storz GmbH & Co, Tuttlingen, Germany) as described previously (Becker et al., 2006; Ernst et al., 2015). Tumours were counted and measured in two dimensions with callipers to calculate the area. Prior to fixation, samples of tumour and adjacent normal epithelium were collected for molecular analysis.

### Histological techniques

Observation of intestinal lineages was performed by staining histological sections (4 µm) with haematoxylin and eosin (H&E; all epithelial cells), phloxine and tartrazine (Paneth cells) and Alcian blue Periodic acid-Schiff stain (goblet cells). To detect cells in the S phase of the cell cycle, mice received an intraperitoneal injection of bromodeoxyuridine (BrdU; 100 mg/kg; Sigma-Aldrich, St Louis, MO, USA) 2 h prior to collection of tissues. Visualisation of apoptosis was performed using the Apop-Tag Peroxidase In Situ Apoptosis Detection Kit (Merck Millipore, Billerica, MA, USA) according to the manufacturer's instructions. Antibodies used to assess cell proliferation (BrdU; BD555624; Life Technologies, Carlsbad, CA, USA), Wnt signalling activation (β-catenin; BD610154; BD Biosciences, San Jose, CA, USA) and GPA33 expression (rabbit polyclonal antibody raised against amino acids 302-319 of mouse GPA33) (Johnstone et al., 2000) were incubated with histological sections and processed as follows. Antigen retrieval was performed by heating to 100°C in 10 mM pH 6 citrate buffer (BDH, Radnor, PA, USA), endogenous peroxidase activity was inhibited with 3% H_2_O_2_ (v/v; BioLab, Lawrenceville, GA, USA) and non-specific antibody binding was blocked using 2% bovine serum albumin (w/v; Sigma-Aldrich, St Louis, MO, USA). Primary antibody binding was followed by incubation with a species-appropriate HRP-conjugated secondary antibody (Dako, Carpinteria, CA, USA), and the signal detected with Liquid Diaminobenzidine substrate Chromogen System (Dako, Carpinteria, CA, USA) prior to counterstaining with haematoxylin. Histological assessment of colitis was performed in a blinded manner using a system comprising crypt loss and inflammation in the LP and submucosa (supplementary material Table S1) (Wirtz et al., 2007).

### Oral tolerance to ovalbumin

On day 0 mice were anaesthetised by intraperitoneal injection with Avertin (0.5 mg/g; Sigma-Aldrich, St Louis, MO, USA) and gavaged with 200 µl sodium bicarbonate (0.75%; BDH Chemicals, Radnor, PA, USA) 15 min prior to immunisation. WT and *Gpa33^−/−^* mice were then gavaged with 200 µl PBS, or 200 µl ovalbumin (OVA) (100 mg/ml, grade III, Sigma-Aldrich, St Louis, MO, USA) in PBS, or 200 µl OVA in combination with cholera toxin B (50 µg/ml; Calbiochem, Billerica, MA, USA) in PBS. On day 7 all mice were injected subcutaneously with a mixture containing 50 µl OVA (1 mg/ml, grade V, Sigma-Aldrich, St Louis, MO, USA) in PBS and 50 µl Freund's Complete Adjuvant (Sigma-Aldrich, St Louis, MO, USA). On day 28, 10 µl of OVA (1 mg/ml, grade V) in PBS was injected subcutaneously into the ear of all mice. Ear thickness was immediately measured with a calliper by a blinded observer (*t*=0 h) and then 24 and 48 h later.

### Statistical data analysis

Data are expressed as mean±s.e.m. unless indicated otherwise. *P*-values were calculated using a two-tailed, unpaired *t*-test with *P<0.05 considered significant and **P<0.01.

## Supplementary Material

Supplementary Material
