# Supplementary material for: Glycoprotein A33 deficiency: a new mouse model of impaired intestinal epithelial barrier function and inflammatory disease
Source: Dis Model Mech. 2015 Aug 1;8(8):805–15. doi: 10.1242/dmm.019935 (PMC4527289; doi:10.1242/dmm.019935)
Supplement: Supplementary Material [file supp_8_8_805__index.html]

Supplementary Material 

# Glycoprotein A33 deficiency: a new mouse model of impaired intestinal epithelial barrier function and inflammatory disease

## DMM019935 Supplementary Material

- Supplementary Material
