# Supplementary material for: Glycoprotein A33 deficiency: a new mouse model of impaired intestinal epithelial barrier function and inflammatory disease
Source: Dis Model Mech. 2015 Aug 1;8(8):805–15. doi: 10.1242/dmm.019935 (PMC4527289; doi:10.1242/dmm.019935)
Supplement: Supplementary Material [file supp_019935_DMM019935supp.pdf]

Supplementary Material

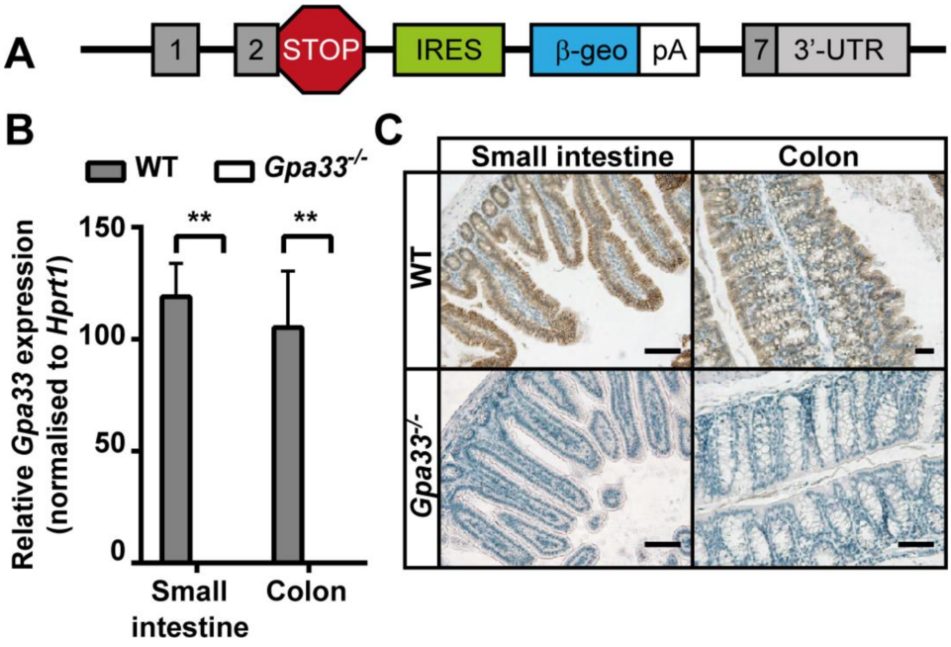

Fig. S1. *Gpa33$^{-/-}$* mice do not express *Gpa33* mRNA or GPA33 protein. (A) The *Gpa33* null allele contains a premature translational stop codon (STOP) in exon 2, and almost all of the coding sequence is replaced with an IRES and *β-geo* gene. (B) *Gpa33* mRNA expression in epithelial cells extracted from the small and large intestine of 10 week old *Gpa33$^{-/-}$* mice is negligible, whereas WT mice exhibit robust expression. Data were normalised to *Hprt1* expression. (C) Representative GPA33 immunohistochemistry in the small and large intestine reveals strong cell surface expression of GPA33 in intestinal epithelial cells of 10 week old WT mice; this is completely absent in *Gpa33$^{-/-}$* mice. Mean ± SEM, n = 3, ** $P < .01$, scale bars 50µm.

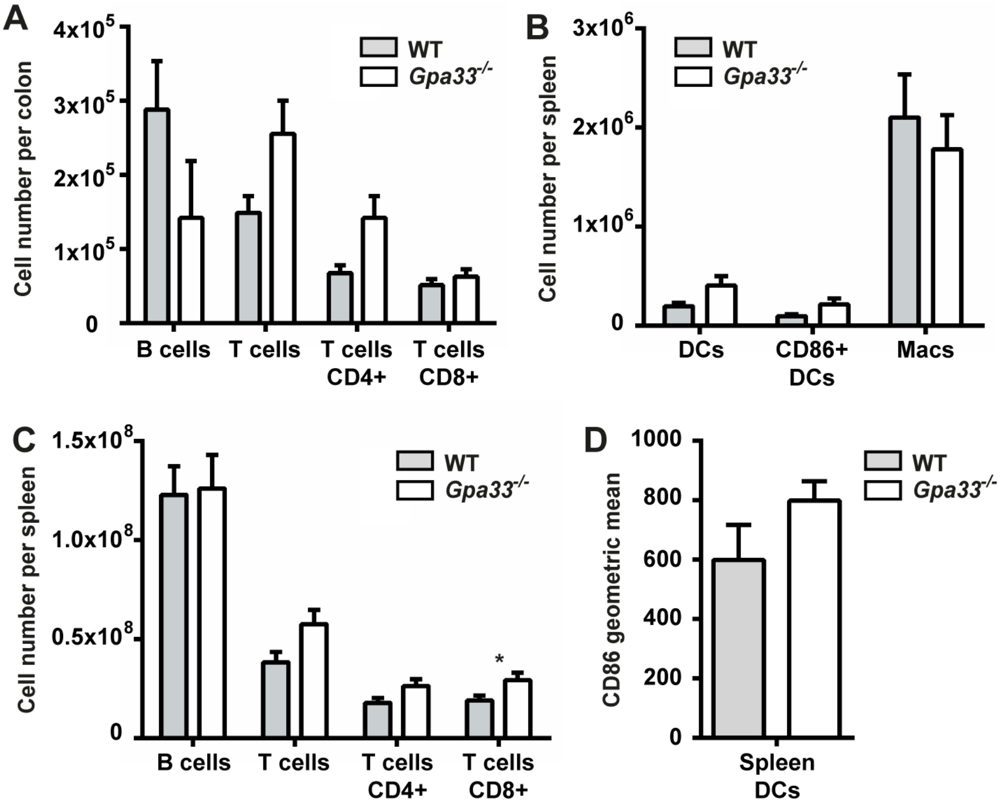

Fig. S2. Flow cytometry analysis of haematopoietic cells within the colon LP and spleen. Single cell suspensions prepared from colon lamina propria (LP) and spleen were analysed by flow cytometry for myeloid and lymphoid populations. Absolute cell numbers for whole colon LP or spleen were calculated using relative cell populations and viable single cell counts. (A) B-cell (Epcam$^-$ CD45$^+$ CD11b$^-$ TCRβ$^-$ B220$^+$) and T-cell (Epcam$^-$ CD45$^+$ CD11b$^-$ TCRβ$^+$ B220$^+$ and CD4$^+$ or CD8$^+$) populations were unchanged in the colon LP of *Gpa33*$^{-/-}$ and WT mice. (B) Dendritic cells (DCs; Epcam$^-$ CD45$^+$ CD11c$^+$ F4/80$^-$ MHC-II$^+$), activated DCs (Epcam$^-$ CD45$^+$ CD11c$^+$ F4/80$^-$ MHC-II$^+$, CD86$^+$) and macrophages (Epcam- CD45$^+$ CD11c$^+$ F4/80$^+$) populations were unchanged in the spleen of *Gpa33*$^{-/-}$ and WT mice. (C) CD8$^+$ T cell (Epcam$^-$ CD45$^+$ CD11b$^-$ TCRβ$^+$ B220$^-$ CD8$^+$) numbers were slightly elevated in the spleen of *Gpa33*$^{-/-}$ and WT mice, whereas B-cell (Epcam$^-$ CD45$^+$ CD11b$^-$ TCRβ$^-$ B220$^+$) and CD4$^+$ T-cell (Epcam$^-$ CD45$^+$ CD11b$^-$ TCRβ$^+$ B220$^-$ and CD4$^+$) numbers were unchanged. Mean ± SEM, n = 6, * $P < .05$.

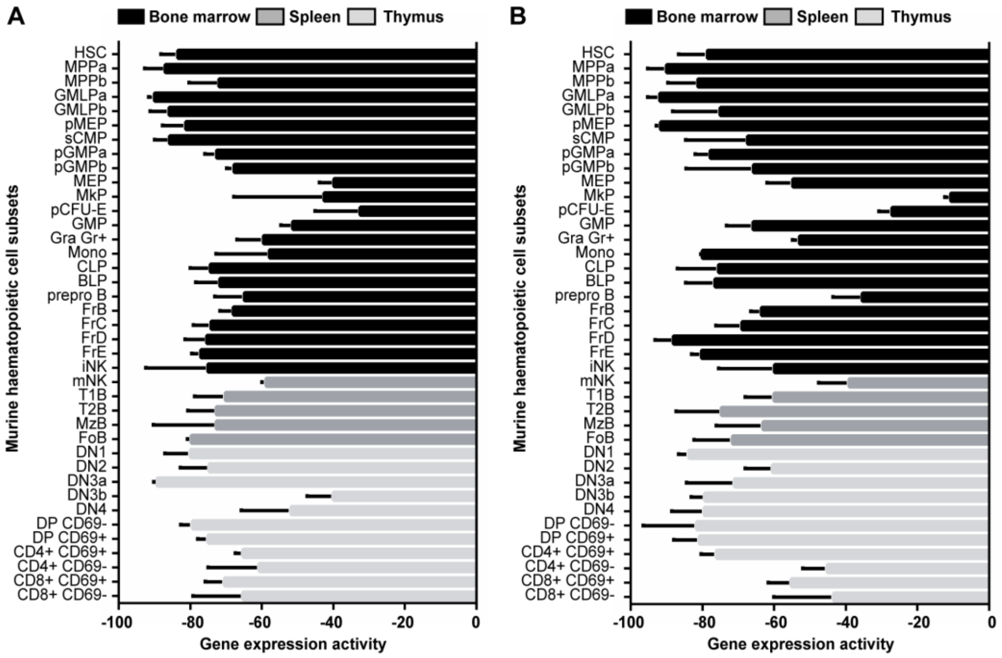

C

Abbreviation	Cell population	Abbreviation	Cell population
HSC	Haematopoietic Stem Cells	FrD	Fraction D B-cell
MPPa	Multi-potent Progenitor subset A	FrE	Fraction E B-cell
MPPb	Multi-potent Progenitor subset A	T1B	T1 B-cell
GMLPa	Granulo/Macrophage/Lymphoid progenitor subset A	T2B	T2 B-cell
GMLPb	Granulo/Macrophage/Lymphoid Progenitor subset B	MzB	Marginal Zone B-cell
pMEP	pre Megakaryocyte/Erythrocyte Progenitor	FoB	Follicular B-cell
sCMP	Strict Common Myeloid Progenitor	iNK	intermediate Natural Killer Cell
pGMPa	preGranulocyte/Macrophage Progenitor subset A	mNK	mature Natural Killer Cell
pGMPb	preGranulocyte/Macrophage Progenitor subset B	DN1	Double Negative T-cell 1
MEP	Megakaryocyte/Erythrocyte Progenitor	DN2	Double Negative T-cell 2
MkP	Megakaryocyte Progenitor	DN3a	Double Negative T-cell 3a
pCFU-E	preCFU-E	DN3b	Double Negative T-cell 3b
GMP	Granlulocyte/Macrophage Progenitor	DN4	Double Negative T-cell 4
Gra Gr+	Granuloctye	DP CD69-	Double Positive CD69- T-cell
Mono	Monocyte	DP CD69+	Double Positive CD69+ T-cell
CLP	Common Lymphoid Progenitor (Ly6d-)	CD4+ CD69+	CD4+ CD69+ T-cell
BLP	Earliest B-lymphoid Progenitor	CD4+ CD69-	CD4+ CD69- T-cell
prepro B	pre pro B cell	CD8+ CD69+	CD8+ CD69+ T-cell
FrB	Fraction B B-cell	CD8+ CD69-	CD8+ CD69- T-cell
FrC B-cell	Fraction C B-cell		

Fig. S3. *Gpa33* is not expressed in haematopoietic populations. Expression of *Gpa33* in 39 haematopoietic populations within the bone marrow, spleen and thymus was analysed using the Gene Expressions Commons (GEXC) Mouse Haematopoiesis Model (dataset GSE34723 (Seita et al., 2012)). This model separated haematopoietic cell populations identified by expression of unique combinations of surface markers with flow cytometry cell sorting. Gene expression was analysed for each cell population on the Affymetrix Mouse Genome 430 2.0 Array (Affymetrix, Santa Clara, CA, USA) microarray platform. Gene expression activity < 0 indicates that *Gpa33* is not expressed in any haematopoietic cell populations. *Gpa33* expression activity was analysed using two independent probesets; (A) probeset 1 and (B) probeset 2. (C) Abbreviations used for cell populations in (A, B). Mean ± SEM, n = 3.

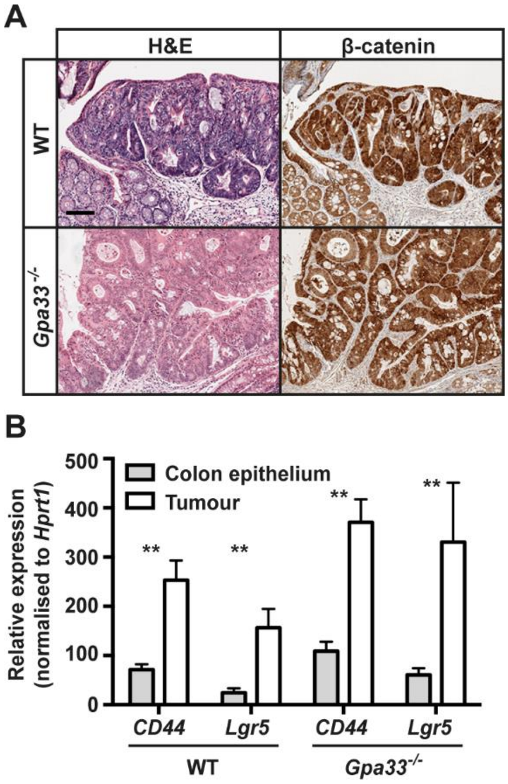

Fig. S4. Both *Gpa33*$^{-/-}$ and WT mice exhibit activation of the WNT pathway in colitis associated tumours. (A) Representative H&E and β-catenin immunohistochemistry of CAC tumours from *Gpa33*$^{-/-}$ and WT mice reveals elevated β-catenin expression within tumours compared to normal epithelium. (B) Relative mRNA expression of WNT pathway target genes *CD44* and *Lgr5* is increased in CAC tumours compared to normal colonic epithelium. Data were normalized to *Hprt1* expression. Mean ± SEM, n = 5-9 for (A, B) and 4-8 for (D), * $P < .05$, ** $P < .01$, scale bar 100μm.

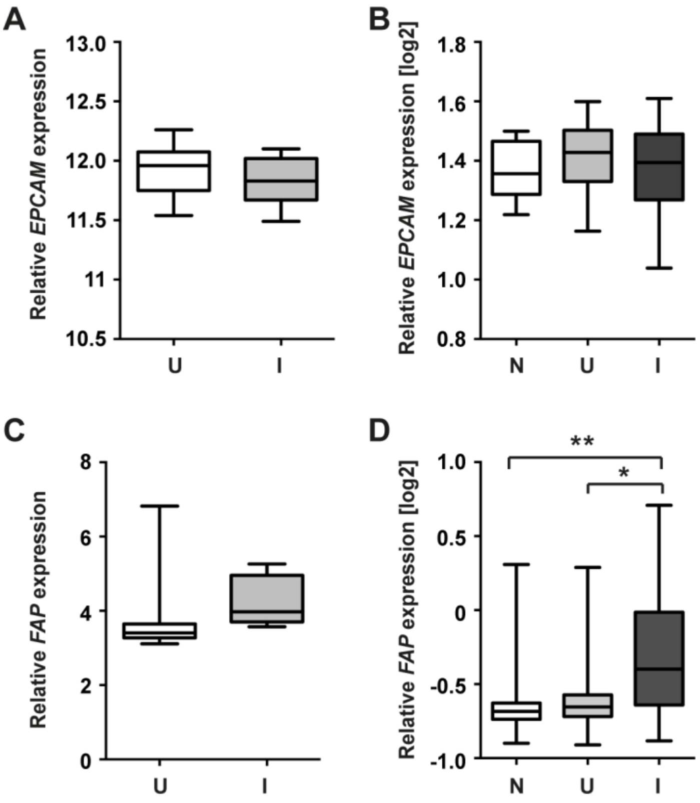

Fig. S5. *EPCAM* and *FAP* expression in the inflamed bowel of IBD patients. Relative *EPCAM* and *FAP* expression in normal (N), uninflamed (U) and inflamed (I) bowel of IBD patients represented as whisker and box plots. (A) *EPCAM* expression is not significantly different in inflamed compared to uninflamed bowel from Crohn's disease patients (uninflamed n = 18, inflamed n = 8; dataset GDS3119) (Olsen et al., 2009) or (B) in inflamed compared to normal and uninflamed bowel from ulcerative colitis patients (normal n = 22, uninflamed n = 15, inflamed n = 19; dataset GDS3268) (Noble et al., 2008). (C) *FAP* expression is not significantly different in inflamed compared to uninflamed bowel from Crohn's disease patients, but is significantly higher in the inflamed compared to the normal and uninflamed tissue from the ulcerative colitis patients (D) * $P < .05$, ** $P < .005$. Relative gene expression values were normalised using (A) Human Genome U133 Plus 2.0 Array Normalisation Controls (Affymetrix) and (B) Stratagene Universal Human Reference (Stratagene).

Table S1. Histological colitis scoring system

The total colitis score comprises epithelial damage and inflammation scores for the proximal, mid and distal colon.

Colitis criteria	Scoring	Tissue compartments
Epithelial damage	0 normal<br>1 hyperplasia<br>2 <50% crypt loss<br>3 >50% crypt loss<br>4 100% crypt loss<br>5 ulceration	Epithelium
Presence of inflammatory cells	0 none<br>1 mild<br>2 moderate<br>3 severe	Mucosa<br>Submucosa<br>Muscle

References

Johnstone, C. N., Tebbutt, N. C., Abud, H. E., White, S. J., Stenvers, K. L., Hall, N. E., Cody, S. H., Whitehead, R. H., Catimel, B., Nice, E. C. et al. (2000). Characterization of mouse A33 antigen, a definitive marker for basolateral surfaces of intestinal epithelial cells. *Am J Physiol Gastrointest Liver Physiol* **279**, G500-10.

Seita, J., Sahoo, D., Rossi, D. J., Bhattacharya, D., Serwold, T., Inlay, M. A., Ehrlich, L. I., Fathman, J. W., Dill, D. L. and Weissman, I. L. (2012). Gene Expression Commons: an open platform for absolute gene expression profiling. *PLoS One* **7**, e40321.
